# FAP-targeting peptide-directed nanoprobes enable tumor microenvironment-activatable MR/NIRF imaging of breast cancer primary tumor and lung metastases

**DOI:** 10.1016/j.mtbio.2026.103064

**Published:** 2026-03-31

**Authors:** Chunting Wang, Jingjing Hu, Yuelin Huang, Yanhong Chen, Ling Zhan, Huanhuan Liu, Defan Yao, Dengbin Wang

**Affiliations:** a Department of Radiology, Xinhua Hospital, Shanghai Jiao Tong University School of Medicine, Shanghai, 200092, China; b Faculty of Medical Imaging Technology, College of Health Science and Technology, Shanghai Jiao Tong University School of Medicine, Shanghai, 200025, China; c Shanghai University of Sport, Shanghai, 200438, China

**Keywords:** Fibroblast activation protein, Breast cancer, Tumor microenvironment, MRI, NIRF

## Abstract

Fibroblast activation protein (FAP), a transmembrane serine protease overexpressed in tumor stroma, has emerged as a dual-purpose biomarker for cancer theranostics. Although FAP-targeting molecular imaging enables precise tumor visualization, its application for microenvironment-responsive detection of breast cancer metastases remains unexplored. To address this, we engineered acidic pH-responsive manganese dioxide (MnO_2_) nanoprobes *via* FAP-targeting peptide-directed biomimetic mineralization. The peptide serves as a biomimetic template, coordinating Mn^2+^ ions with a macrocyclic ligand to direct oxidative deposition under physiological conditions. This design integrates two key functions: FAP-specific targeting and acid-triggered MnO_2_ nanoprobes releasing Mn^2+^ for T_1_-weighted MR contrast enhancement; Cy7 fluorescence increase achieving tumor-to-background ratio in NIRF imaging. In orthotopic and metastatic breast cancer models, the nanoprobes enabled high-resolution visualization of primary tumors and lung metastases as small as a diameter of 0.3 mm. In addition, the novel molecular imaging probe demonstrated favorable biosafety and efficient renal clearance, supporting the potential for clinical translation. This work establishes a tumor stroma-targeted activatable platform advancing metastasis-sensitive diagnosis and providing a blueprint for microenvironment-responsive theranostics.

## Highlights

• Novel acid-responsive nanoprobes were engineered for specific FAP targeting.
• The nanoprobes enable sensitive T_1_-weighted MRI and NIRF imaging of TNBC.
• Acid-triggered Mn^2+^ release enhances T_1_-weighted MRI within tumor microenvironment.
• The system successfully achieved *in vivo* detection of TNBC lung metastases.

## Graphical abstract

**Figure:**
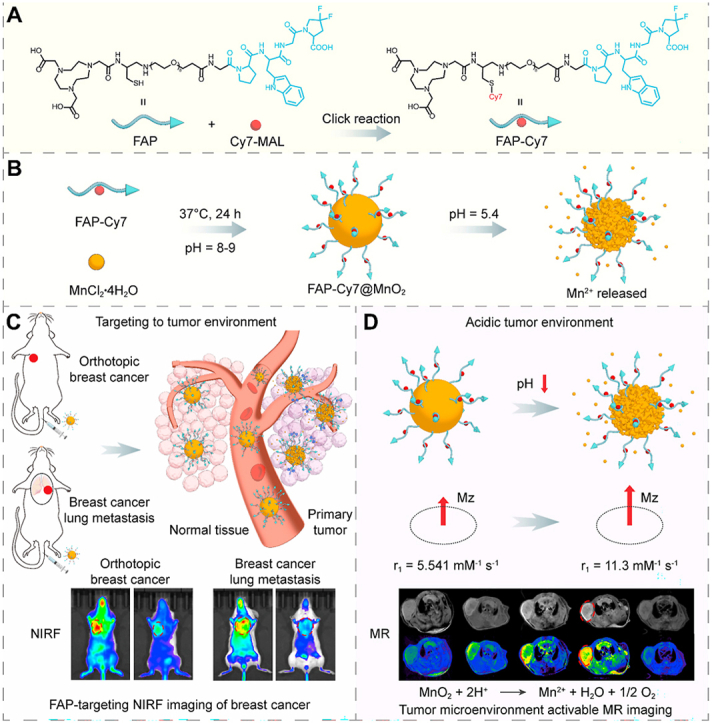

## 1 Introduction

Breast cancer is the most common cancer and the leading cause of cancer-related mortality among women, with its incidence continuing to rise. In 2022, there were 2.3 million new cases diagnosed, accounting for 11.6% of all cancer cases. Thus, it continues to pose a major public health challenge. Thus, it continues to pose a major public health challenge [1,2]. Triple-negative breast cancer (TNBC), regarded as the most aggressive subtype of breast cancer, is characterized by a high proliferative index, an increased risk of recurrence, poor overall survival rates, and the highest incidence of metastasis among the various breast cancer subtypes [3]. Its tendency to spread early highlights the critical importance of early detection through screening and prompt intervention, as these measures can significantly improve survival rates and clinical outcomes [4].

In recent years, advancements in cancer research have highlighted the critical role of the tumor microenvironment (TME) in cancer progression. The dynamics of tumor growth, metastasis, and invasion are closely linked to the TME [5,6]. The acidic TME, which arises from complex interactions between breast cancer cell metabolism, vascular abnormalities, and tumor-associated mechanisms, suppresses the activity of key tumor-infiltrating immune cells, such as T cells and natural killer (NK) cells [7,8]. As a result, considerable research efforts have focused on targeting and modulating the acidic TME, particularly through the development of nanoprobes that are responsive to these acidic conditions [9,10]. Building on the significance of the TME, fibroblast activation protein (FAP) has emerged as a critical biomarker in breast cancer, particularly in TNBC [[11], [12], [13], [14], [15], [16], [17]]. FAP is highly overexpressed in the stromal compartment of TNBC, in contrast to its minimal or absent expression in normal tissues, making it a specific and reliable biomarker for this aggressive subtype of breast cancer [18]. Previous researches have shown that FAP plays a key role in tumor progression, metastasis, and treatment resistance, all of which are characteristic features of TNBC. Given its elevated expression in most malignant tumors, FAP has become a promising target for both diagnostic imaging and therapeutic interventions in TNBC [19,20].

Imaging plays a crucial role in the non-invasive diagnosis and treatment assessment of breast cancer. The primary imaging modalities include mammography, ultrasound (US), magnetic resonance imaging (MRI), and ^18^F-fluorodeoxyglucose positron emission tomography/computed tomography (^18^F-FDG PET/CT) [[21], [22], [23], [24]]. Among the imaging techniques, MRI serves as a highly sensitive and non-invasive tool for breast cancer detection and characterization, due to high-resolution capabilities for soft tissue visualization, absence of ionizing radiation, and ability to obtain multi-paramet-data [25]. While MRI detects breast cancer with 89% sensitivity, its specificity is lower at 79% [26]. Gadolinium-based contrast agents, such as gadolinium DOTA meglumine, are commonly used in MRI but have limitations, including rapid renal filtration and poor tissue targeting [27]. These limitations hinder the full potential of MRI in targeted disease imaging. In contrast, NIRF imaging offers rapid response times and high sensitivity [28,29]. However, its *in vivo* application is restricted by limited tissue penetration depth [30,31]. Accordingly, by combining the complementary strengths of MRI and NIRF imaging, through fragments-targeting imaging probes delivery, this approach allows for highly sensitive, spatially resolved, and selective tumor visualization [32,33].

Recently, novel radiotracers based on fibroblast activation protein inhibitors (FAPI) have been developed, showing promising potential for future applications [[34], [35], [36]]. However, concerns about radioactivity and cost have hampered broader clinical adoption and routine use of FAP-targeted PET probes. Consequently, there is a growing need for non-radioactive FAP-targeted molecular imaging strategies, such as peptide-guided nanoparticle probes, to facilitate wider translation and advance cancer diagnostics. Advances in peptide-nanoparticle conjugates (PNCs) have demonstrated significant potential in enhancing the specificity and precision of therapeutic platforms [37,38]. The integration of peptides provides highly selective targeting capabilities, reducing off-target effects, while nanoparticles enhance both the imaging and therapeutic functionalities of the conjugates. Additionally, PNCs can be engineered for optimal biocompatibility and stability, improving their pharmacokinetic profiles and facilitating their clinical translation [39]. Manganese dioxide (MnO_2_) has garnered significant attention as a distinctive TME-responsive nanoprobe [40]. Numerous studies have demonstrated that MnO_2_ nanostructures can interact with H^+^ within the TME, subsequently releasing paramagnetic Mn^2+^ to facilitate T_1_-weighted MRI (T_1_-MRI) for targeted tumor detection [[41], [42], [43]]. Furthermore, the integration of MnO_2_ nanostructures with NIRF imaging components, along with the incorporation of TME-targeted fragments, facilitates the realization of TME multimodal imaging, thereby enhancing the precision of diagnostic outcomes [44,45].

**Scheme 1:**
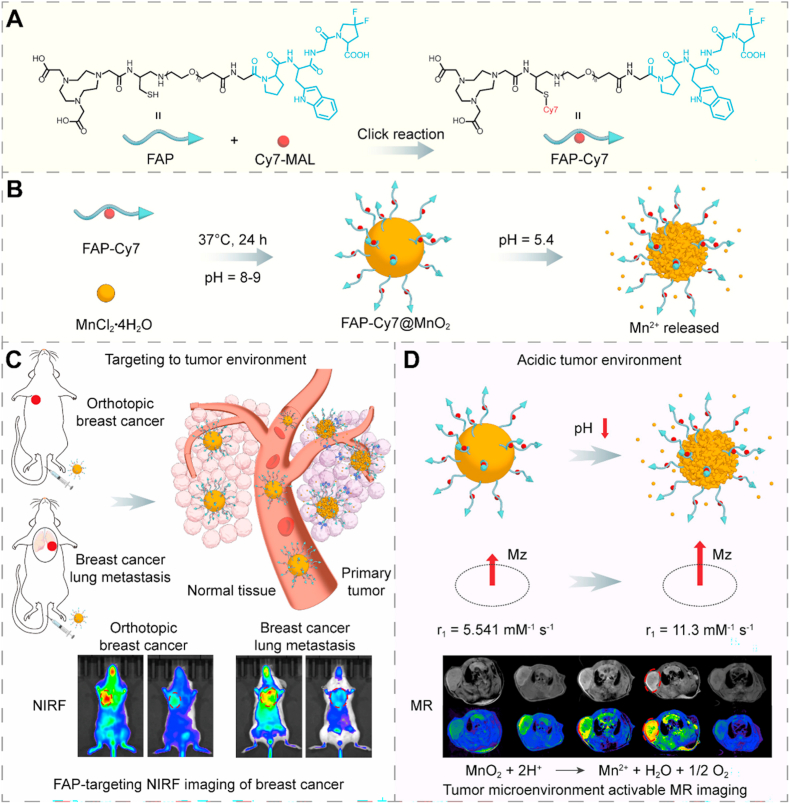
Schematic illustration of the synthesis and imaging mechanisms of FAP-Cy7@MnO_2_. (A and B) Chemical structure of FAP-Cy7 and the synthesis process for MnO_2_ nanoparticles conjugated with FAP-targeting peptides. (C) Imaging mechanism for FAP-targeting NIRF imaging. (D) Mechanism of MR imaging activation in response to the acidic TME.

To address the diagnostic challenges, we developed an acidic pH-responsive FAP-Cy7@MnO_2_ nanoprobe through FAP-targeting peptide-directed biomimetic mineralization. This approach utilized macrocyclic ligand-conjugated peptides that coordinate Mn^2+^ ions, facilitating oxidative deposition into MnO_2_ nanostructures under physiological conditions. The resultant nanoprobe incorporates FAP-specific targeting through peptide recognition and TME-activatable dual-mode imaging. It achieves acid-triggered Mn^2+^ release for enhanced T_1_-weighted MRI contrast and Cy7 fluorescence increase, thereby attaining a high tumor-to-background ratio in NIRF imaging. Unlike PET, which relies on radioactive tracers, MRI provides superior soft tissue contrast and spatial resolution without exposing patients to ionizing radiation. This combination of excellent anatomical detail and inherent safety makes MRI particularly advantageous for precise tumor localization and longitudinal monitoring. FAP-targeted MRI enables precise molecular-level tumor localization and quantification of FAP activity. Additionally, NIRF imaging offers enhanced tissue penetration and clearer imaging [46,47]. In this study, we combine FAP-targeted imaging with MnO_2_ nanoparticles for MR and NIRF dual-modal imaging, enhancing tumor visualization through prolonged circulation times and pH-responsive Mn^2+^ release. Although this study does not directly compare with FAPI-based PET tracers or other FAP-MRI agents, the combination of FAP-targeting with MnO_2_ nanomaterials presents a promising approach for future clinical applications. In orthotopic breast cancer models, the probe enabled high-resolution visualization of primary tumors and lung metastases. The probe exhibited excellent biosafety and efficient renal clearance, supporting its potential for clinical translation. This work pioneers a dual-modality theranostic platform enabling metastasis detection through TME-responsive activation, establishing a new design paradigm for stroma-targeted molecular imaging (Scheme 1).

## 2 Methods and materials

### 2.1 Materials

Purified water was obtained using the Millipore filtration system. Gd-DOTA was purchased from Adamas-beta Co., Ltd (Shanghai, China). The BCA protein concentration assay kit, enhanced CCK8 assay kit, D-Luciferin potassium salt, Lyso-Tracker, Mito-Tracker, anti-GAPDH antibody, HRP goat anti-rabbit IgG(H + L), Cy3-conjugated goat anti-rabbit IgG(H + L), and DAPI solution were all obtained from Beyotime (Shanghai, China). The anti-FAP antibody was sourced from Signal way Antibody (USA, 54991). Cell culture reagents, including fetal bovine serum (FBS), RPMI-1640, Dulbecco's modified Eagle's medium (DMEM), 0.25% trypsin, and Penicillin-Streptomycin, were sourced from Gibco.

### 2.2 Synthesis of FAP-Cy7@MnO_2_

The FAP-targeting peptide was synthesized and purified following our previously established research, incorporating additional modifications. In summary, the peptide was synthesized using Fmoc-based SPPS and subsequently purified through preparative high-performance liquid chromatography (HPLC). As demonstrated in our earlier research, this peptide exhibits a high binding affinity for FAP-overexpressing cancer-associated fibroblasts (CAFs) and demonstrates significant accumulation in both primary gastric tumors and peritoneal metastatic lesions [48]. Building on this FAP-targeting peptide, we developed a novel FAP peptide-modified manganese dioxide nanoparticle (FAP-Cy7@MnO_2_). Following peptide synthesis, the sulfhydryl group of the peptide was conjugated with Cy7-maleimide (Cy7-Mal) *via* a thiol-maleimide click reaction. The resultant product (FAP-Cy7) underwent dialysis in ultrapure water for 24 h and was subsequently lyophilized to produce the final FAP-targeting fluorescent probe.

Subsequently, FAP-Cy7@MnO_2_ nanoparticles were synthesized *via* a mild two-step method: (1) MnCl_2_ solution was added dropwise into an aqueous solution containing the FAP-Cy7 probe under stirring. (2) The subsequent reaction was initiated by maintaining the mixture pH at 8–9 through the addition of 0.1 M NaOH:

$$2{\text{MnCl}}_{2}+4\text{NaOH}+O_{2}={\text{MnO}}_{2}+4\text{NaCl}+4H_{2}O$$

The reaction proceeded overnight under stirring at room temperature, followed by 30 min of ultrasonication to facilitate nanoparticle formation, yielding FAP-Cy7-modified MnO_2_ nanoparticles (FAP-Cy7@MnO_2_).

### 2.3 Material characterizations

A suite of characterization techniques was employed to analyze the synthesized materials. Morphology and hydrodynamic size were assessed using transmission electron microscopy (TEM, FEI Tecnai F20) and dynamic light scattering (DLS, Malvern Nano ZS), respectively, while elemental distribution was visualized *via* elemental mapping. Material composition and phase were investigated through XPS (Thermo K-alpha) and XRD (X'Pert PRO MPD). Finally, UV-vis absorption spectra were acquired on an Agilent Cary 100 spectrophotometer at room temperature.

### 2.4 Cell culture

The murine 4T1, HC11 (mouse mammary epithelial cells), MDA-MB-231 and MDA-MB-468 cell lines were obtained from the Cell Bank of the Chinese Academy of Sciences, Shanghai, China. These cell lines were maintained in RPMI-1640 or DMEM medium, supplemented with 10% fetal bovine serum and 1% penicillin-streptomycin solution, under controlled conditions of 37 °C in a humidified atmosphere containing 5% CO_2_.

### 2.5 Detection of protein expression at the cellular level

The expression levels of FAP in 4T1 and HC11 cells were confirmed through western blot analysis and anti-FAP immunofluorescence staining. Similarly, the expression levels of FAP in MDA-MB-231 and MDA-MB-468 cells were validated using anti-FAP immunofluorescence staining. These analyses utilized an anti-FAP antibody, with subsequent incubation using HRP-conjugated goat anti-rabbit IgG(H + L) at a dilution of 1:250 for Western blotting and Cy3-conjugated goat anti-rabbit IgG(H + L) at a dilution of 1:200 for immunofluorescence staining. *In vitro* FAP was extracted from the 4T1 and HC11 cell lines using RIPA lysis buffer, with a protease inhibitor concentration of PMSF: RIPA at a ratio of 1:100.

### 2.6 Cell viability assay

To assess the cytotoxic effects of FAP-Cy7@MnO_2_, 4T1 and HC11 cell lines were cultured in 96-well plates. The cells were then exposed to a range of FAP-Cy7@MnO_2_ concentrations, from 0 to 128 μM, for a period of 24 h. Additionally, we seeded 4T1 and HC11 cells into 96-well plates and incubated them at different time points with 128 μM FAP-Cy7@MnO_2_ to observe the toxicity response of the probes under high-concentration conditions. Following this exposure, 10 μL of enhanced CCK-8 solution was added to each well, and the cells were incubated at 37 °C for an additional hour. Absorbance was measured at 450 nm using a Thermo Fisher Scientific Spectramax Microplate Reader to determine relative cell viability. Additionally, 4T1 cells were seeded in a six-well plate at a density of 1 × 10^6^ cells/mL. Upon reaching approximately 60% confluence, a solution of FAP-Cy7@MnO_2_ at a concentration of 128 μM was introduced. Following a 6-h incubation period, the cells underwent three gentle washes with phosphate-buffered saline (PBS). Subsequently, the cells were stained with calcein-AM/PI for 15 min and visualized using microscopy.

### 2.7 Mitochondrial membrane potential (MMP) assessment

4T1 cells were seeded into 24-well plates (5 × 10^4^ cells per well) and incubated overnight. The cells were then treated according to the same protocols outlined in the **Cell viability assay** section. Following this, the cells were stained using the enhanced mitochondrial membrane potential assay kit with JC-1 (Beyotime Ltd, China) according to the manufacturer's instructions. Finally, the cells were observed under a fluorescence microscope (OLYMPUS, Japan). In polarized mitochondria (normal cells), JC-1 accumulates and forms aggregates, emitting high red fluorescence (Ex/Em 585/590 nm). In depolarized mitochondria, JC-1 remains in its monomeric form, displaying green fluorescence (Ex/Em 514/529 nm).

### 2.8 Cellular uptake and colocalization analysis

To investigate the intracellular localization of FAP-Cy7@MnO_2_, 4T1 cells were cocultured with FAP-Cy7@MnO_2_ for 2 h, followed by incubation with Mito-Tracker and Lyso-Tracker for 30 min. After a 5-min DAPI counterstain and two rinses with PBS, cells were imaged on a Leica TCS SP5 confocal microscope. Subsequent image analysis was performed in ImageJ to determine fluorescence intensity and organelle co-localization coefficients.

### 2.9 Animal experiment

Female BALB/c mice, aged 5-6 weeks, were procured from the Shanghai Laboratory Animal Center and maintained in a pathogen-free environment. All experimental procedures involving animals were conducted under the guidelines sanctioned by the Ethics Committee of Xinhua Hospital, affiliated to Shanghai Jiao Tong University School of Medicine (Approval No. XHEC-F-2024-014). Each mouse received an injection of 1.0 × 10^6^ 4T1 cells, suspended in 100 μL of PBS, administered between the abdominal mammary pads. For the establishment of the lung metastasis model, 1.0 × 10^6^ 4T1 cells suspended in 100 μL of PBS were injected *via* tail vein into each mouse. The mice were then monitored for the development of metastases, and subsequent imaging and analysis were performed to assess tumor progression. MRI and NIRF imaging were performed on separate groups of animals from the same batch, rather than in a single animal. This approach allows us to leverage the unique strengths of both imaging modalities, ensuring that MRI and NIRF together provide a more comprehensive evaluation of tumor characteristics.

### 2.10 4T1 in orthotopic tumor fluorescence imaging and MRI

Mice were divided into two groups (n = 3 per group): the target group, designated as FAP-Cy7@MnO_2_, and the inhibitor group, designated as FAPI + FAP-Cy7@MnO_2_. The FAPI + FAP-Cy7@MnO_2_ group was administered *via* tail vein injection with an FAP-targeting peptide at a concentration twice that of the FAP-Cy7@MnO_2_ probe, 3 h prior to the injection of FAP-Cy7@MnO_2_. This pre-treatment effectively blocks the FAP-binding sites, preventing the nanoprobes from accumulating at the tumor site and ensuring a proper control comparison. Following the intravenous administration of the probe (100 μL, 0.5 mM), *in vivo* NIRF imaging was conducted at specified intervals of 0.1, 0.5, 1, 1.5, 2, 4, and 24 h using the IVIS Imaging System (PerkinElmer). At 24 h post-injection, mice were euthanized for *ex vivo* NIRF imaging of tumors and major organs (λex/λem = 650/670 nm).

The orthotopic implantation of 4T1 breast cancer mouse models was divided into three groups: the FAP-Cy7@MnO_2_ group, the FAPI + FAP-Cy7@MnO_2_ group, and the Gd-DOTA group (n = 3). Each group was injected *via* tail vein with 200 μL of FAP-Cy7@MnO_2_ (10 mM) or an equal volume of Gd-DOTA. The FAPI + FAP-Cy7@MnO_2_ group was pre-injected with an FAP-targeting peptide at a concentration twice that of the FAP-Cy7@MnO_2_ probe, 3 h prior to the injection of FAP-Cy7@MnO_2_. T_1_-weighted RARE and T_1_-mapping RARE axial images were obtained using a 9.4T Bruker BioSpec system at baseline and subsequently at 0.5h, 1h, 4h, and 24h post-injection. The imaging parameters were as follows: field of view (FOV) of 3.5 × 3.0 cm, slice thickness of 1 mm, slice gap of 1 mm, repetition time (TR) of 500 ms, echo time (TE) of 4.85 ms, number of averages = 2, and image size of 200 × 172. For the T_1_ measurement, 1/T_1_ values were calculated for each region of interest (ROI) in the tumor and muscle. The following parameters were used in the calculation: SNR (signal-to-noise ratio) = SI tumor/SD air. CNR (contrast-to-noise ratio) = (SI tumor - SI muscle)/SD air. These values were used to evaluate the relative enhancement of the tumor signals over time, providing quantitative assessment of the imaging contrast.

### 2.11 Pharmacokinetic and biodistribution analysis

To evaluate the pharmacokinetics and organ distribution characteristics of FAP-Cy7@MnO_2_, inductively coupled plasma mass spectrometry (ICP-MS) was used to measure manganese concentrations in various organs and blood at different time points after injection. Mice were divided into two groups: the FAP-Cy7@MnO_2_ group and the FAPI + FAP-Cy7@MnO_2_ control group. Manganese distribution in the organs was analyzed for both groups, with a particular focus on assessing manganese concentration in the kidneys and comparing the differences between the two groups to evaluate renal clearance. Blood samples were collected from the FAP-Cy7@MnO_2_ group, and were collected at 5, 10, 15, 30, 45, 60, 90, and 120 min post-injection for ICP-MS quantification of manganese content. By monitoring the changes in blood manganese concentration over time, the pharmacokinetic profile was determined, revealing the probe's half-life, clearance rate, and organ accumulation characteristics.

### 2.12 Fluorescence imaging, bioluminescence of lung metastases

Lung metastatic Balb/c mice were administered an intraperitoneal injection of D-luciferin potassium salt 10 min before imaging, followed by an intravenous injection of the FAP-Cy7@MnO_2_ probe (100 μL, 0.5 mM). Bioluminescence and NIRF imaging were conducted within the 650 nm excitation and 670 nm emission range utilizing the IVIS imaging system. NIRF imaging of organs and tumors *in vitro* was performed in mice after euthanasia at 30 min, 1 h, and 2 h post-injection, respectively, to determine probe uptake and distribution.

### 2.13 *In vivo* biocompatibility

Balb/c mice were randomly assigned to either a control group or a group treated with FAP-Cy7@MnO_2_ *via* tail vein injection. Upon sacrifice, key organs (including the heart, liver, spleen, lungs, and kidneys) were collected and processed for hematoxylin and eosin (H&E) staining and immunohistochemical (IHC) examination. Blood was also drawn from all mice by ocular extraction for comprehensive hematological and biochemical evaluations.

### 2.14 Hemolysis test

Blood was collected from healthy mice and subsequently mixed with PBS to prepare a 2% erythrocyte suspension. To assess hemolysis, a 2% erythrocyte suspension was prepared with increasing concentrations of FAP-Cy7@MnO_2_ and incubated at 37 °C for 2 h. The samples were then centrifuged (5000 rpm, 10 min, 4 °C), and the hemolytic rate was evaluated based on the ultraviolet absorbance of the supernatant at 540 nm. PBS at pH 7.4 served as a negative control, while a 2% erythrocyte lysate was employed as a positive control.

### 2.15 Statistical analysis

All data are presented as mean ± standard deviation (SD). Statistical comparisons were performed using a two-tailed Student's t-test or two-way ANOVA, with post-hoc tests as appropriate. A p-value of ∗p < 0.05 was considered statistically significant, ∗∗p < 0.01 was highly significant, ∗∗∗p < 0.001 was very highly significant, and ∗∗∗∗p < 0.0001 was considered extremely significant. Significance levels are denoted as follows: ∗p < 0.05, ∗∗p < 0.01, ∗∗∗p < 0.001, and ∗∗∗∗p < 0.0001.

## 3 Results and discussion

### 3.1 Preparation and characterization of FAP-targeting nanoprobes

**Fig. 1:**
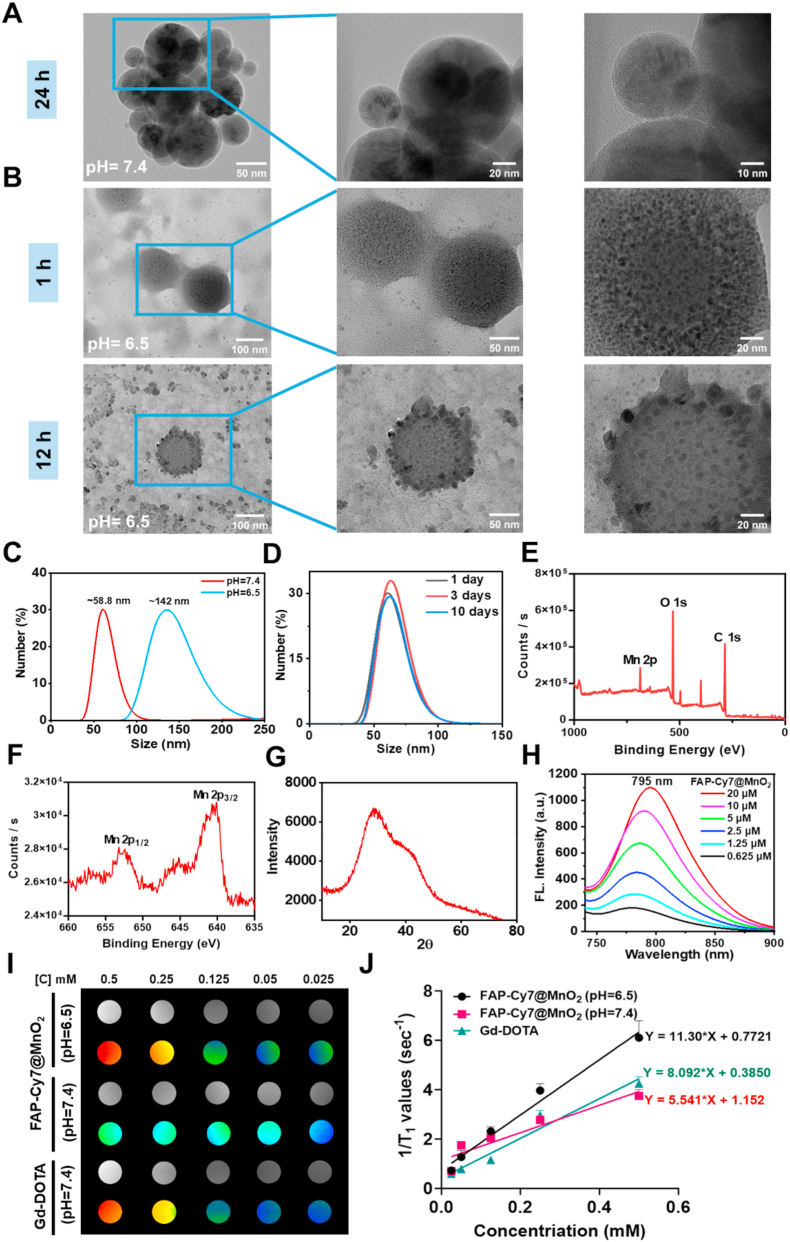
Characterization of FAP-Cy7@MnO_2_ nanoparticles. (A-B) TEM images of FAP-Cy7@MnO_2_ at pH 7.4 and pH 6.5, showing changes at different time points. (C) DLS analysis of FAP-Cy7@MnO_2_ at different pH level, illustrating size variations. (D) DLS analysis of FAP-Cy7@MnO_2_ at various time points under room temperature conditions. (E-F) X-ray photoelectron spectroscopy XPS spectra confirming the composition of FAP-Cy7@MnO_2_. (G) XRD analysis of FAP-Cy7@MnO_2_, confirming its amorphous nature. (H) Fluorescence spectra of FAP-Cy7@MnO_2_ nanoparticles at varying concentrations, demonstrating their optical properties. (I) MR images of FAP-Cy7@MnO_2_ (at pH 6.5 and 7.4) and Gd-DOTA at different concentrations (0.025, 0.05, 0.125, 0.25, and 0.5 mM) in aqueous solution, showing varying imaging intensities. (J) Longitudinal relaxation rate (1/T_1_) plotted against concentrations, indicating a linear relationship and highlighting the enhanced relaxivity at pH 6.5.

FAP-targeting peptides were synthesized using the standard Fmoc-based SPPS technique. Characterization data, including those from preparative HPLC and mass spectrometry, have been previously reported in our group's research [48]. For NIRF imaging, Cy7-mal was conjugated to the peptides *via* a thiol-maleimide reaction. Additionally, to enable MR imaging, a mild two-step synthesis was employed to conjugate the FAP-targeting peptides with MnO_2_ nanoparticles, resulting in the FAP-Cy7@MnO_2_ nanoprobe [49]. TEM was employed to examine the morphological changes of FAP-Cy7@MnO_2_ nanoparticles under neutral and acidic conditions. As shown in Fig. 1A, TEM analysis revealed that under neutral pH (pH 7.4), the FAP-Cy7@MnO_2_ nanoparticles exhibited a spherical structure with a grain size of approximately 50–60 nm, confirming the successful synthesis of the FAP-MnO_2_ conjugate. To evaluate the long-term stability of FAP-Cy7@MnO_2_ nanoparticles, we conducted experiments over 10 days after synthesis. The nanoparticles were dispersed in deionized water, RPMI-1640 cell culture media, or normal saline at a concentration of 1 mM. DLS measurements were taken on days 1, 3, and 10. As shown in Fig. 1D, the particle size remained stable around 58.8 nm throughout the 10-day period. In addition, TEM images in Fig. S1A and B confirm that there were no major changes in the morphology or particle size. Fig. S1C and D further show that the particle size did not change significantly when the nanoparticles were incubated in RPMI-1640 media or normal saline for 10 days. These results indicate that FAP-Cy7@MnO_2_ nanoparticles are highly stable for at least 10 days.

However, as the incubation time was extended, the nanoparticles underwent acid-induced degradation under acidic conditions (pH 6.5). This caused their structure to collapse and resulted in the formation of particles larger than 100 nm. These findings suggest that the acidic TME promotes the breakdown of the nanoparticles, facilitating the release of Mn^2+^ ions (Fig. 1B and C) [50]. Elemental mapping analysis (Fig. S2) further confirmed the effective coupling between the FAP-targeting peptide and MnO_2_ nanoparticles, as both neutral and acidic conditions showed the presence of Mn, O, and F elements. The evolution of particle size was further evaluated at different time points, with DLS and TEM analyses conducted at 24 and 48 h, as shown in Fig. S3 and Fig. S4. TEM images taken after 24 h of incubation (Fig. S3A) reveal that the core of the nanoprobes was further eroded by the acidic conditions, causing the nanoparticle structure to break down. This acid corrosion process released Mn^2+^ ions, which then underwent self-nucleation, forming smaller manganese-containing nanoparticles that were dispersed around the original probe [51]. By 48 h (Fig. S3B), TEM images and DLS measurements (Fig. S4) show that only nanoparticles smaller than 10 nm remained, indicating that the nanoprobes were nearly completely acid-eroded, with most of the manganese ions having been released into the solution. To investigate the pH-dependent release of manganese ions, FAP-Cy7@MnO_2_ nanoparticles were incubated at 37 °C under different pH conditions and analyzed by ICP-MS. After 48 h, the cumulative release of manganese ions at pH 6.5 reached 14.2 μg/L, significantly higher than the 2.6 μg/L observed at pH 7.4. This data clearly indicates that MnO_2_ nanoparticles degrade more rapidly in the acidic environment, leading to an accelerated release of Mn^2+^ ions (Fig. S5) [52].

Additionally, the formation of MnO_2_ was confirmed by X-ray photoelectron spectroscopy (XPS). The XPS spectrum revealed two distinct peaks corresponding to the Mn (IV) 2p_1_/_2_ and 2p_3_/_2_ energy levels, indicating the predominance of Mn (IV) oxidation state and confirming the presence of MnO_2_ in the nanoparticles (Fig. 1E and F) [53,54]. This confirmation validates the chemical state of manganese in the probe, which is crucial for its intended function, especially the controlled release of Mn^2+^ ions in response to the acidic TME. X-ray diffraction (XRD) analysis further confirmed the predominantly amorphous nature of MnO_2_ (Fig. 1G). We then obtained the fluorescence emission spectrum (Fig. 1H) and ultraviolet-visible (UV-Vis) absorption spectrum (Fig. S6) of FAP-Cy7@MnO_2_ in PBS at pH 7.4. Both spectral measurements showed a characteristic absorption maximum around 750 nm, confirming the expected optical properties of the material. Furthermore, FAP-Cy7@MnO_2_ exhibited a strong fluorescence emission peak centered at 795 nm, confirming its near-infrared (NIR) optical properties. This emission profile is advantageous for biomedical imaging, as it provides enhanced tissue penetration depth and reduced background autofluorescence. In acidic conditions (pH = 6.5), the fluorescence spectrum of FAP-Cy7@MnO_2_ nanoparticles revealed an increase in fluorescence intensity, which can be attributed to the degradation of MnO_2_. Under neutral or mildly acidic conditions, MnO_2_ remains stable and does not exhibit significant changes. In acidic conditions (pH = 6.5), the fluorescence spectrum of FAP-Cy7@MnO_2_ nanoparticles showed an increase in fluorescence intensity, likely due to the degradation of MnO_2_. While MnO_2_ remains stable under neutral or mildly acidic conditions, in the acidic TME, MnO_2_ degrades, releasing Mn^2+^. This degradation not only reduces the quenching effect on Cy7 fluorescence but also may alter the probe's charge state and surface properties, further enhancing the fluorescence signal [[55], [56], [57], [58]]. These changes in optical behavior contribute to the stronger fluorescence observed, confirming the probe's responsiveness to acidic conditions and its potential for improved tumor imaging (Fig. S7). Notably, a concentration-dependent bathochromic shift in the fluorescence emission maximum was observed. This redshift is likely due to molecular aggregation, which results from increased intermolecular interactions at higher concentrations, leading to the formation of J-aggregate-like species with altered photophysical properties [59,60].

Additionally, the paramagnetic properties of FAP-Cy7@MnO_2_ (pH 6.5/7.4) and Gd-DOTA were evaluated by measuring their longitudinal relaxation time (T_1_) using a Bruker 9.4 T MRI scanner [61]. Fig. 1I and J shows the T_1-_weighted imaging (T_1_-WI) signals of FAP-Cy7@MnO_2_ at pH levels 6.5 and 7.4, as well as those of Gd-DOTA, across various concentrations. As the Mn concentration increased, the T_1_ MRI signal intensity and T_1_ mapping results for FAP-Cy7@MnO_2_ at pH 7.4 remained relatively stable. In contrast, under acidic conditions (pH 6.5), the FAP-Cy7@MnO_2_ probe signals were effectively activated, showing significantly higher intensity compared to both the pH 7.4 group and the non-targeted Gd-DOTA group at all concentrations, as depicted in Fig. 1I. Quantitative analysis of T_1_-mapping RARE images revealed a linear relationship between the proton longitudinal relaxation rate (r_1_) and the concentration of the probes (Fig. 1J). Based on the slope, the T_1_ relaxivity of FAP-Cy7@MnO_2_ at pH 6.5 was calculated to be 11.3 mM^−1^s^−1^, which is approximately twice as high as that at pH 7.4 (5.541 mM^−1^s^−1^) and also exceeds the value for the clinical agent Gd-DOTA (8.092 mM^−1^s^−1^). Since 1/T_1_ is a crucial parameter for enhancing MRI contrast, these findings suggest that the FAP-Cy7@MnO_2_ nanoparticles has the potential to improve MRI contrast in tumor tissues.

### 3.2 High expression of FAP in TNBC

**Fig. 2:**
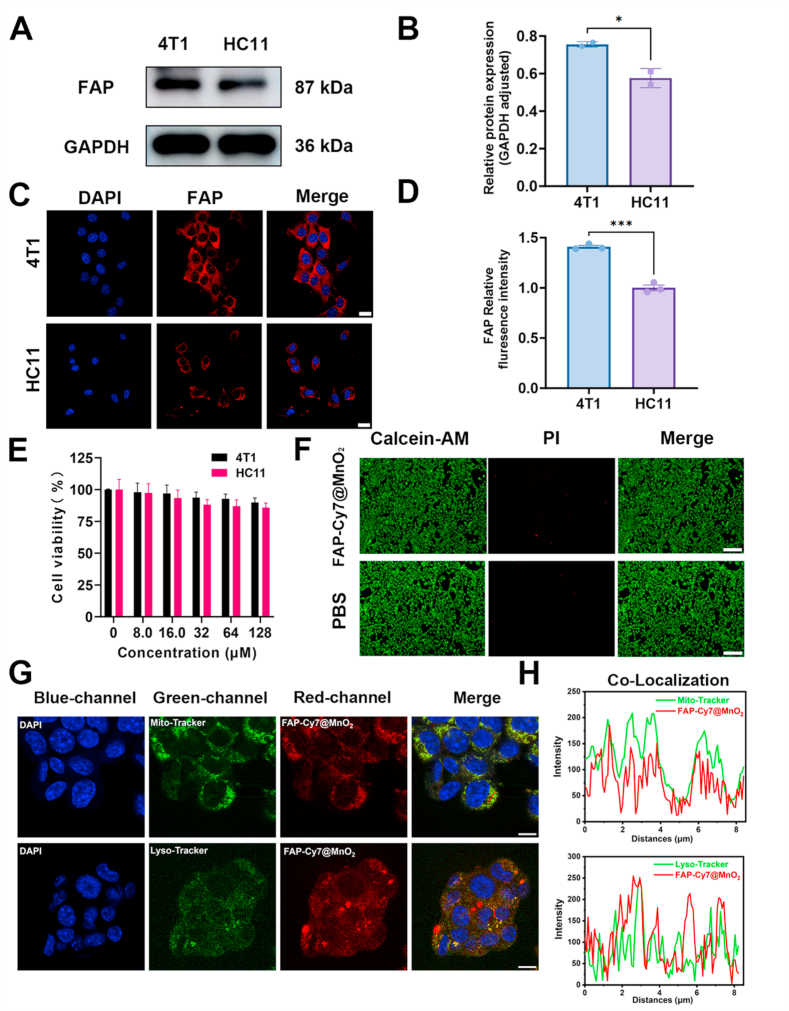
FAP Expression and FAP-Cy7@MnO_2_ Co-localization Study. (A-B) Western blot analysis of FAP protein expression in 4T1 and HC11 cells. (C-D) Representative immunofluorescence images and quantification of FAP expression in 4T1 and HC11 cells. Scale bar: 8 μm. (E) Cell viability assay of 4T1 and HC11 cells incubated with different concentrations of FAP-Cy7@MnO_2_ nanoprobes. (F) Live/Dead staining (Calcein-AM/PI) of 4T1 cells treated with 128 μM FAP-Cy7@MnO_2_. Scale bar: 100 μm. (G) Confocal microscopy images of 4T1 cells incubated with 12.5 μM FAP-Cy7@MnO_2_ (red) and co-stained with Lyso-Tracker Green (lysosomes), Mito-Tracker Green (mitochondria), and DAPI (nuclei). (H) Co-localization analysis of FAP-Cy7@MnO_2_ with organelle-specific trackers. Excitation/Emission: FAP-Cy7@MnO_2_ (λex = 633 nm, λem = 730-790 nm); Trackers (λex = 504 nm, λem = 570 nm); DAPI (λex = 340 nm, λem = 488 nm). Scale bar: 8 μm. (For interpretation of the references to colour in this figure legend, the reader is referred to the Web version of this article.)

Given the well-established upregulation of FAP in malignant tumors such as TNBC, its expression at the cellular level was assessed in this study using western blot analysis and immunofluorescence staining [[62], [63], [64], [65]]. Western blot results revealed significantly elevated FAP expression levels in 4T1 TNBC cells compared to murine mammary epithelial cells (HC11) (Fig. 2A and B). Immunofluorescence staining further confirmed the predominant membrane localization of FAP, with high surface expression observed in 4T1 cells and minimal expression in HC11 cells (Fig. 2C and D). After confirming that FAP is highly expressed in 4T1 breast cancer cells compared to normal cells, we also selected other TNBC cell lines, MDA-MB-231 and MDA-MB-468, and further validated the significant overexpression of FAP in TNBC through immunofluorescence experiments (Fig. S8). Collectively, these findings highlight FAP as a promising target for TNBC [66].

### 3.3 Cellular uptake and cytotoxicity studies

To evaluate the safety and targeting efficiency of the FAP-targeting probe, the cytotoxicity of FAP-Cy7@MnO_2_ at various concentrations was first assessed *in vitro*. As shown in Fig. 2E, the FAP-Cy7@MnO_2_ nanoprobes were well-tolerated by cells at all tested concentrations, highlighting their favorable biosafety profile. Further biosafety assessment was conducted using live/dead co-staining in 4T1 cells with Calcein-AM (live, green) and propidium iodide (dead, red). Notably, cells treated with 128 μM FAP-Cy7@MnO_2_ exhibited green fluorescence intensity comparable to that of untreated controls, with minimal red fluorescence indicating minimal cell death (Fig. 2F). These findings collectively demonstrate the high biosafety of the probe at biologically relevant concentrations. Studies have shown that manganese ions, when present at sufficient concentrations, can serve as an ideal adjuvant for activating the immune system against tumors [67,68]. Manganese enhances cGAS sensitivity and catalytic efficiency, activating the STING pathway, leading to the release of type I interferons, improved tumor antigen presentation, T cell activation, and M1 TAM polarization, thereby promoting antitumor immunity [52]. Regarding the toxicity of the probe, our results suggest that Mn^2+^ exhibits low toxicity to both tumor cells and normal cells, indicating good biosafety. The CCK-8 assay (Fig. 2E) shows that at a concentration of 128 μM, the survival rate of both 4T1 and HC11 cells remains above 85%, indicating that the released Mn^2+^ concentration is insufficient to induce significant cytotoxicity at this early stage. Further CCK-8 experiments (Fig. S9) were performed after incubating 4T1 and HC11 cells with the probe for 24, 48, and 96 h. After 24 h of incubation, the viability of both 4T1 and HC11 cells remained above 80%, suggesting minimal cytotoxicity at this time point. However, as the incubation time increased, the viability of both cell types gradually decreased. This decline in viability suggests that the prolonged release of Mn^2+^ over time gradually enhances the cytotoxic effect, likely due to the continuous release of Mn^2+^. Additionally, both tumor and normal cells may possess antioxidant mechanisms that help mitigate the potential toxicity of Mn^2+^, contributing to the observed selective toxicity. This selective cytotoxicity is consistent with the known principle that metal ion toxicity is influenced by both the concentration of ions and the exposure time [69,70].

To investigate the subcellular localization of FAP-Cy7@MnO_2_, 4T1 breast cancer cells were co-incubated with the nanoprobes for 2 h, followed by staining with Mito-Tracker (for mitochondria) or Lyso-Tracker (for lysosomes) for 30 min, and counterstaining with DAPI (for the nucleus) for 5 min. As shown in Fig. 2G, significant overlap (yellow regions) was observed between FAP-Cy7@MnO_2_ and Mito-Tracker signals, indicating mitochondrial localization. Quantitative analysis using ImageJ revealed a high Pearson's colocalization coefficient of 0.617 for FAP-Cy7@MnO_2_ with Mito-Tracker, compared to a significantly lower coefficient of 0.383 with Lyso-Tracker (Fig. 2H). Upon cellular internalization, FAP-Cy7@MnO_2_ nanoprobes are initially taken up by endocytosis and subsequently localize to acidic endosomes and lysosomes. In the acidic environment of the lysosome, MnO_2_ nanoparticles may partially dissolve, releasing Mn^2+^ ions and altering the surface charge of the nanoprobes, enabling them to escape the lysosome. This escape mechanism is likely facilitated by the pH gradient across the lysosomal membrane, where the acidic pH inside promotes protonation of the MnO_2_ surface, destabilizing the nanoprobe and allowing it to move to the cytosol or mitochondria. Once released into the cytosol, FAP-Cy7@MnO_2_ nanoprobes are directed to mitochondria, likely due to the incorporation of mitochondrial-targeting peptides, which enhance the probe's affinity for the organelle. This mitochondrial targeting not only enhances imaging but also ensures that released Mn^2+^ ions are confined to the mitochondria, enabling more precise tumor imaging and potential therapy while minimizing interference with other cellular components [71,72].

To investigate the influence of FAP-Cy7@MnO_2_ nanoprobes on mitochondrial function, we utilized the MitoProbe JC-1 Assay Kit to assess changes in mitochondrial membrane potential (MMP) in 4T1 cells under various treatments. JC-1 aggregates exhibit red fluorescence and form in mitochondria with high MMP, while JC-1 monomers show green fluorescence in depolarized mitochondria of dying cells. The results demonstrated that after treatment with FAP-Cy7@MnO_2_ nanoprobes, the red fluorescence (aggregates) in the cells was significantly enhanced, indicating no notable loss of MMP, with no significant difference compared to the control group (Fig. S10). This suggests that FAP-Cy7@MnO_2_ nanoprobes did not significantly impact the mitochondrial membrane potential in 4T1 cells, and no signs of cell death were observed.

### 3.4 *In vivo* FAP-targeting NIRF imaging of breast cancer

**Fig. 3:**
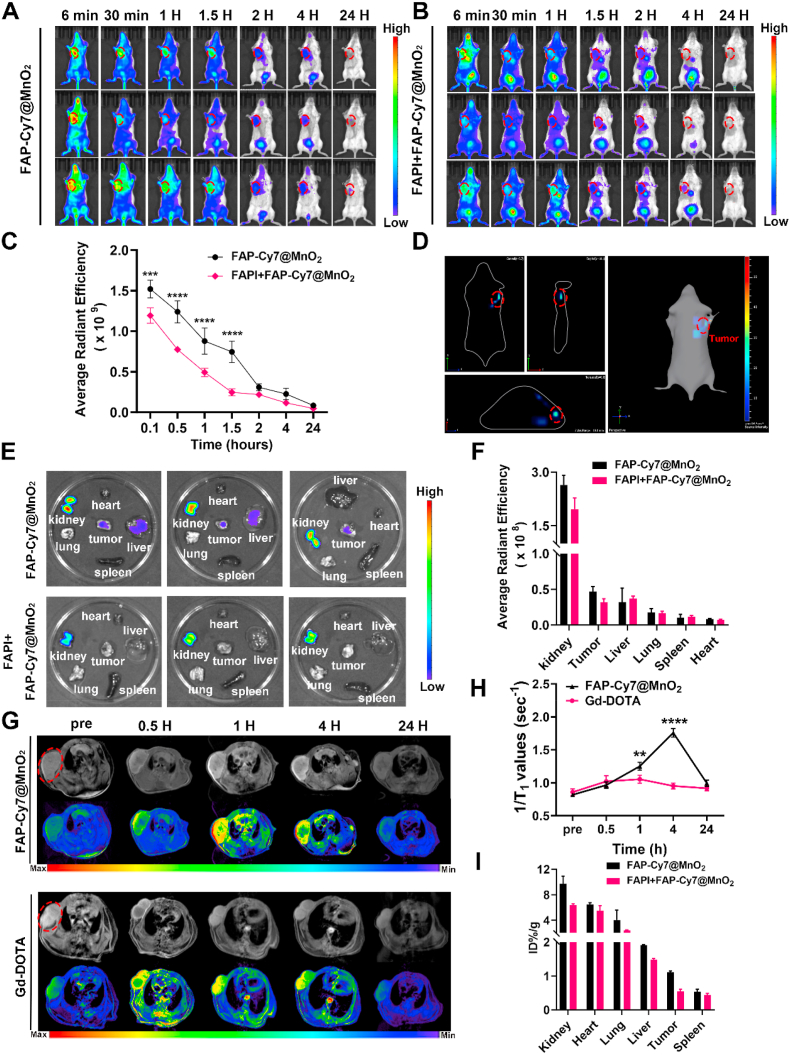
FAP-Targeting NIRF and MR Imaging in Orthotopic Breast Cancer-Bearing Mice. (A-B) *In vivo* NIRF imaging of two groups of 4T1 tumor-bearing mice after intravenous injection of FAP-Cy7@MnO_2_ and FAPI + FAP-Cy7@MnO_2_, respectively. Tumors are delineated by dashed lines in all images, captured under the same imaging conditions. (C) Quantification of tumor accumulation in the two groups. (D) 3D NIRF imaging of the FAP-Cy7@MnO_2_ group at 2 h post-injection (∗∗∗*p* < 0.001, ∗∗∗∗*p* < 0.0001). (E) NIRF imaging and (F) quantitative analysis of probe accumulation in excised organs and tumors 24 h after injection of FAP-Cy7@MnO_2_ and FAPI + FAP-Cy7@MnO_2_. (G) Representative *in vivo* T_1_-weighted MR images and (H) the corresponding quantitative time-dependent changes in 1/T_1_ relaxation rate following the injection of FAP-Cy7@MnO_2_ or Gd-DOTA (∗∗*p* < 0.01, ∗∗∗∗*p* < 0.0001). (I) *In vivo* biodistribution study of FAP-Cy7@MnO_2_ and FAPI + FAP-Cy7@MnO_2_.

*In vivo* fluorescence imaging was conducted to evaluate the tumor retention and biodistribution of FAP-Cy7@MnO_2_ in 4T1 tumor-bearing mice. The mice were randomly divided into two groups (n = 3): the targeted group (FAP-Cy7@MnO_2_) and the non-targeted group (FAPI + FAP-Cy7@MnO_2_, containing the FAP inhibitor). To monitor the real-time biodistribution, fluorescence imaging was performed on mice after tail vein injection of either FAP-Cy7@MnO_2_ or FAPI + FAP-Cy7@MnO_2_ using a dedicated small-animal imaging system. As shown in Fig. 3A–C, the fluorescence intensity in the tumor region peaked at 6 min post-injection for both groups, confirming the effective NIRF imaging capabilities of the nanoprobes. Notably, mice in the FAP-Cy7@MnO_2_ group exhibited significantly higher fluorescence signals throughout the imaging period compared to the FAPI + FAP-Cy7@MnO_2_ group. Additionally, as shown in Fig. S11 and 6 minutes after the injection of all probes, the signal-to-noise ratio (SNR) and contrast-to-noise ratio (CNR) of the FAP-Cy7@MnO_2_ group were 1.29 times and 1.26 times higher, respectively, compared to the FAPI + FAP-Cy7@MnO_2_ control group. This indicates that FAP-Cy7@MnO_2_ effectively accumulates at TNBC tumor sites *via* targeted enrichment, resulting in a higher tumor-to-background ratio. Three-dimensional (3D) imaging further enhanced visualization of the tumor site. As shown in Fig. 3D and Video 1, 3D imaging of the FAP-Cy7@MnO_2_ group 2 h post-injection provided a comprehensive view of the tumor. At 24 h post-injection, *in vitro* imaging and organ analysis revealed low fluorescence signal intensity in both groups, with FAP-Cy7@MnO_2_ exhibiting higher fluorescence intensity than FAPI + FAP-Cy7@MnO_2_. Except for the kidneys, which showed some fluorescence, other organs displayed minimal fluorescence, indicating that FAP-Cy7@MnO_2_ nanoparticles were primarily metabolized and cleared through the kidneys (Fig. 3E and F).

Supplementary data related to this article can be found online at https://doi.org/10.1016/j.mtbio.2026.103064

The following are the Supplementary data related to this article:

Multimedia component 1 Multimedia component 1

### 3.5 *In vivo* FAP-targeting MRI of breast cancer

Subsequently, we evaluated the efficacy of FAP-Cy7@MnO_2_ as a contrast agent for MRI in detecting tumors in mice implanted with 4T1 cells. Non-targeted Gd-DOTA and FAPI + FAP-Cy7@MnO_2_ were used for comparison. T_1_ RARE imaging was performed at 0.5, 1, 4, and 24 h following intravenous administration of Gd-DOTA and FAP-Cy7@MnO_2_ (Fig. 3G and Fig. S12). After probe administration, both groups showed enhanced tumor signals, with signal intensity progressively decreasing over time and returning to baseline levels by 24 h. As shown in Fig. 3H, the tumor signal intensity reached its peak at 4 h post-injection of FAP-Cy7@MnO_2_. The FAP-Cy7@MnO_2_ group exhibited a signal intensity approximately 1.80-fold higher than the control group. Additionally, as depicted in Fig. S12 and 4 hours after injection of all probes, the SNR and CNR of the FAP-Cy7@MnO_2_ group were 1.66 times and 2.62 times higher, respectively, compared to the FAPI + FAP-Cy7@MnO_2_ control group. These MRI results strongly corroborated the fluorescence imaging findings, confirming the probe's excellent targeting specificity toward orthotopic breast tumors. By correlating the Mn^2+^ release profile with changes in signal intensity over time, we show that even small differences in Mn^2+^ release can result in significant increases in imaging signal, especially within the 4-h MRI imaging window. To evaluate the pharmacokinetics and organ distribution characteristics of FAP-Cy7@MnO_2_, we used ICP-MS to quantify manganese concentrations in various organs and blood at different time points after injection. As shown in Fig. 3I, the FAP-Cy7@MnO_2_ group exhibited higher manganese accumulation in the kidneys, followed by the heart and lungs, indicating that the probe was primarily cleared through the renal system. The FAPI + FAP-Cy7@MnO_2_ control group, where FAP-targeting sites were blocked, showed significantly lower manganese accumulation in the tumor and organs, highlighting the critical role of FAP-targeting for specific accumulation in the tumor tissue.

In the blood pharmacokinetics analysis (Fig. S13), the FAP-Cy7@MnO_2_ group demonstrated a rapid decrease in manganese concentration over time, with the blood concentration decreasing to approximately 0.5% ID/g after 120 min, suggesting efficient metabolism and clearance from the circulation. This result indicates the probe's relatively short half-life and rapid systemic clearance, with the majority of the probe accumulating in the kidneys for excretion.

Overall, these findings demonstrate that FAP-Cy7@MnO_2_ exhibits effective tumor targeting and efficient renal clearance, with the FAPI + FAP-Cy7@MnO_2_ control group showing significantly reduced tumor accumulation. This confirms the specific targeting of the probe to FAP-positive tumor tissues, enhancing its potential for imaging and therapeutic applications.

Therefore, FAP-Cy7@MnO_2_ enables both NIRF and MR imaging. The FAP-targeted group showed stronger tumor accumulation compared to the FAPI inhibitor group. The enhanced tumor accumulation in the FAPI inhibitor group is likely due to the enhanced permeability and retention (EPR) effect, which facilitates passive accumulation through leaky tumor vasculature. While the FAPI inhibitor blocks FAP binding, the observed accumulation highlights the EPR effect. In contrast, the FAP-targeted group shows more specific tumor accumulation driven by the targeting mechanism, emphasizing the dominant role of FAP-targeted binding in precise tumor targeting. Additionally, fluorescence imaging at 6 min post-injection showed maximal tumor targeting of FAP-Cy7@MnO_2_, reflecting the initial accumulation of the nanoparticles due to their FAP-specific binding. In contrast, MRI at 4 h post-injection displayed peak tumor signal enhancement, corresponding to the release of Mn^2+^ ions from the nanoprobes *via* a pH-responsive mechanism. This release process is triggered by the acidic characteristics of TME, resulting in a prolonged MRI signal enhancement effect. Fluorescence imaging maintained high brightness from 0 to 4 h, while the pH-triggered release mechanism allows MRI signal enhancement to persist over a longer period. For early tumor diagnosis, fluorescence imaging at 6 min post-injection is most suitable, while MRI at 4 h provides superior contrast for detailed imaging of deeper tumors.

### 3.6 *In vivo* FAP-targeting NIRF imaging of breast cancer lung metastases

**Fig. 4:**
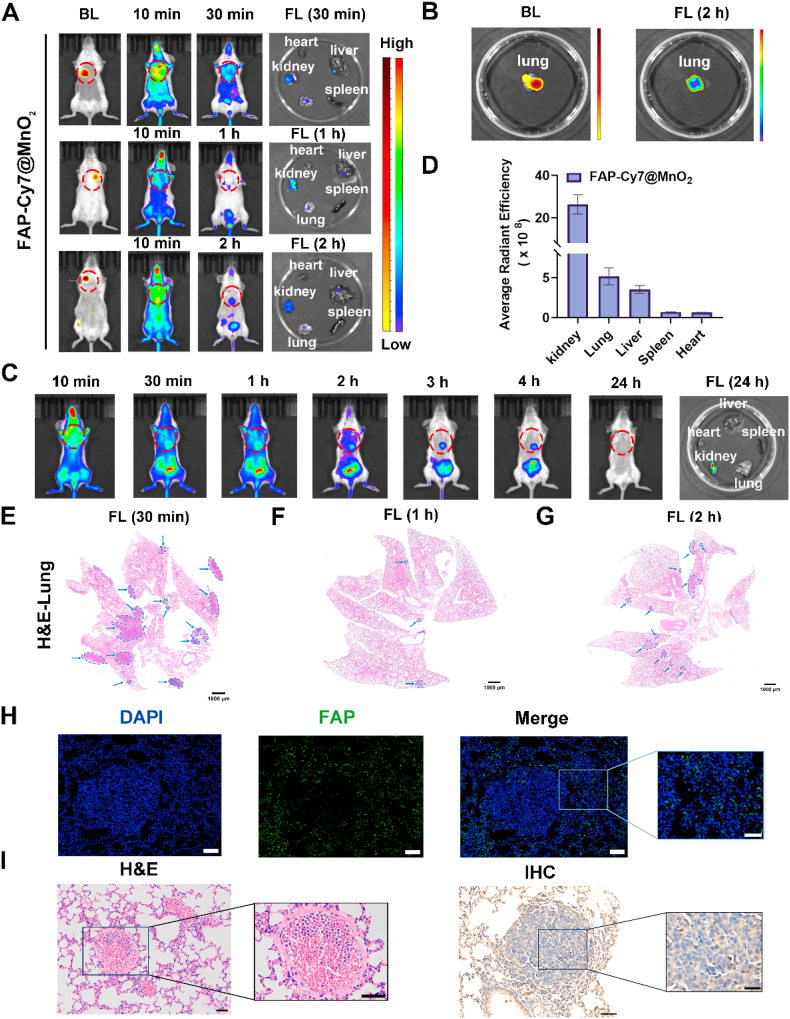
FAP-Targeting NIRF Imaging in a Breast Cancer Lung Metastasis Model. (A-B) *In vivo* and *in vitro* bioluminescence (BL) and NIRF imaging of 4T1-luc-bearing mice injected with FAP-Cy7@MnO_2_. (C) *In vivo* NIRF imaging at multiple time points in 4T1-luc mice following administration of FAP-Cy7@MnO_2_. (D) Quantification of fluorescence intensity in dissected organs and tumors of mice 24 h post-injection of FAP-Cy7@MnO_2_. (E-G) H&E staining of pulmonary metastatic lesions at different imaging time points. Blue arrows indicate. Scale bars: 1000 μm. (H-I) Anti-FAP immunohistochemical, immunofluorescence, and H&E staining of lung metastases. Scale bars: 50 μm. (For interpretation of the references to colour in this figure legend, the reader is referred to the Web version of this article.)

Following NIRF/MR imaging in orthotopic breast cancer models, the FAP-Cy7@MnO_2_ probe-targeted group displayed significantly higher fluorescence signals compared to the inhibitor (FAPI-Cy7@MnO_2_) and control (Gd-DOTA) groups, respectively. Additionally, the probe-targeted group showed predominant renal metabolism within 24 h, highlighting the efficient targeting and favorable metabolic rate of the FAP-targeting probes. The lung, known for its distinct anatomical structure that efficiently captures circulating tumor cells, is a common site for cancer metastasis. To model breast cancer lung metastasis, mice were intravenously injected with 4T1-luc cells *via* the tail vein. Subsequently, D-fluorescein potassium salt was injected intraperitoneally into mice with induced breast cancer lung metastases, and the localization of lung metastatic lesions was assessed using *in vivo* bioluminescence imaging. Ten minutes after intravenous administration of the FAP-Cy7@MnO_2_ probe, a hyper-fluorescence signal in the lungs was observed, correlating with the bioluminescence signal (Fig. 4A). Following euthanasia, *in vitro* imaging of the lesions revealed an increase in both bioluminescence and fluorescence signals, with the bioluminescence signals matching the fluorescence signals (Fig. 4B and Fig. S14). A 24-h monitoring study showed metabolic outcomes similar to those observed in the orthotopic tumor model. Additionally, fluorescence intensity at the tumor site peaked 10 min post-injection, followed by gradual metabolism *via* the kidneys (Fig. 4C and D). To further validate the sensitivity of FAP-Cy7@MnO_2_ in detecting lung metastases, we performed histological analysis of lung tissue at 30 min, 1 h, and 2 h post-injection, as shown in Fig. 4(E–G). H&E staining was used to identify metastatic nodules, and we observed a strong correlation between the fluorescence signal intensity in the lungs and both the number and size of metastatic lesions. As the number of metastatic nodules increased, the fluorescence intensity also increased, with larger nodules showing stronger fluorescence. This indicates that the fluorescence intensity is directly related to the size and number of the metastatic lesions. Notably, the smallest detected nodule, with a diameter of 0.3 mm, further demonstrates the high sensitivity of FAP-Cy7@MnO_2_ in detecting small lung metastases. Additionally, histological analyses, including H&E staining and IHC and immunofluorescence assessments, revealed elevated FAP expression in metastatic breast cancer tissues within the lung (Fig. 4H and I). These findings support the use of our probe for early-stage detection of metastatic disease.

### 3.7 Biodistribution and biosafety

**Fig. 5:**
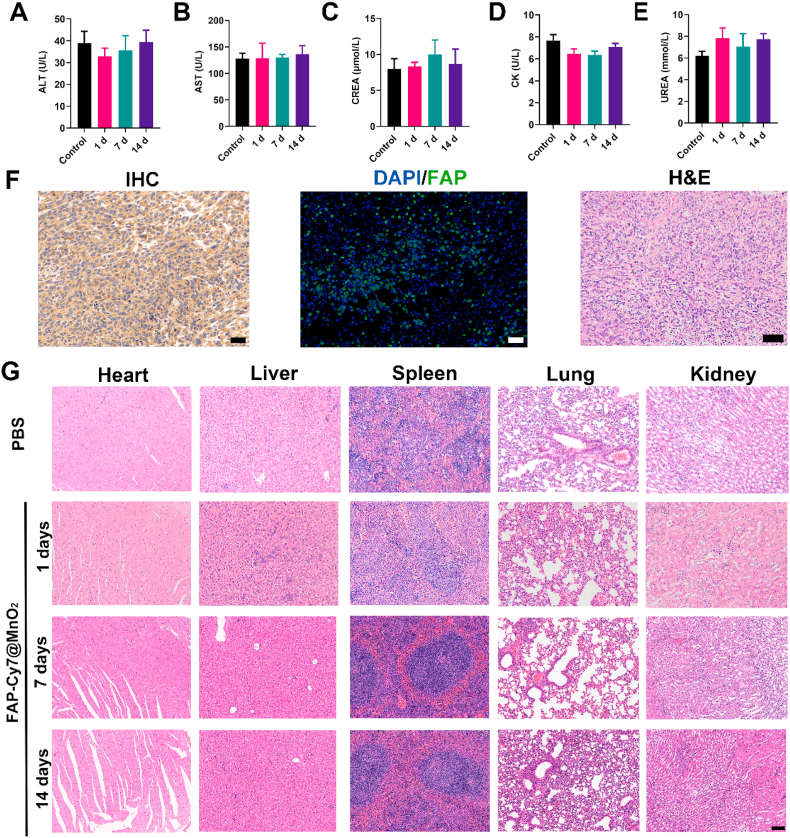
Biosafety Analysis of FAP-Cy7@MnO_2_. (A-E) Blood biochemistry assays (liver and kidney function tests) in healthy mice 1, 7, 14 days after intravenous injection of FAP-Cy7@MnO_2_. (F) Anti-FAP immunohistochemistry, immunofluorescence, and H&E staining of 4T1 tumors in an orthotopic breast cancer model. Scale bar: 20 μm. (G) H&E staining of excised organs 24 h, 7 days, and 14 days after injection of PBS or FAP-Cy7@MnO_2_. Scale bar: 50 μm.

The *in vivo* biocompatibility of the FAP-Cy7@MnO_2_ probe was further assessed at 1, 7, and 14 days following tail vein injection. Hematological analyses, including measurements of alanine aminotransferase (ALT), aspartate aminotransferase (AST), creatinine (CREA), creatine phosphokinase (CK), and urea nitrogen (UREA), were performed to evaluate liver and kidney function. The results showed that all values remained within the normal range and did not exhibit significant differences when compared to the control group (Fig. 5A–E), indicating that the probe does not cause substantial hepatotoxicity or nephrotoxicity at the dosages used. The potential for red blood cell damage was assessed by incubating red blood cells with varying concentrations of FAP-Cy7@MnO_2_ (1.5 mM, 1.0 mM, 0.5 mM, 0.25 mM, and 0.125 mM) and measuring the hemolysis percentage. As shown in the graph (Fig. S15), the probe induced negligible hemolysis at all concentrations, with levels similar to the PBS control, indicating that the FAP-Cy7@MnO_2_ probe is safe and does not cause significant red blood cell damage. Histological analysis of excised tissue from the orthotopic breast cancer tumor confirmed high FAP expression in the tumor sections (Fig. 5F). IHC staining of major organs revealed no significant FAP expression (Fig. S16), further supporting the high target specificity of the probe. Additionally, H&E staining of major organs was conducted to evaluate potential toxicity. The results showed that, at 1 day, 7 days, and 14 days post-injection in tumor-bearing mice, the cellular morphology of the major organs remained unchanged, with no significant pathological alterations detected (Fig. 5G). These findings suggest that FAP-Cy7@MnO_2_ does not induce significant adverse effects on normal tissues, supporting its potential as a safe imaging agent.

## 4 Conclusion

In this study, we successfully developed a pH-activatable, FAP-targeted bimodal probe for MR and NIRF imaging of breast cancer and its metastases. The probe demonstrated robust FAP-specific binding both *in vitro* and *in vivo*, enabling the controlled release of manganese ions in response to the acidic TME. The combination of MRI and NIRF imaging effectively addressed the limitations of single-modality imaging, providing enhanced diagnostic capabilities. The system's biosafety was confirmed through negligible cytotoxicity, normal blood parameters, and unaltered organ histology, further supporting its clinical potential. By combining FAP targeting with TME responsiveness, the probe significantly reduced off-target signals and markedly improved the tumor-to-background ratio. This strategy not only enhances diagnostic accuracy but also provides a foundation for designing targeted therapeutic agents.

However, we recognize that the path to clinical translation for a complex nanoprobe like this is long and fraught with challenges, including large-scale manufacturing, regulatory approval, and ensuring reproducibility in clinical settings. The synthesis of the probe relies on Fmoc-SPPS and multi-step MnO_2_ deposition, which, while well-established, may present challenges in terms of batch-to-batch reproducibility and yield. To address these, future work will focus on optimizing the synthesis process to improve scalability and ensure consistency. Additionally, we acknowledge that improving the dispersibility of these nanoparticles is crucial for enhancing their clinical applicability and safety. Therefore, we plan to explore surface modification strategies, such as PEGylation, to improve colloidal stability, prevent aggregation, and enhance circulation time in biological fluids. Furthermore, optimizing the synthesis conditions to control particle size and surface charge will be explored to reduce aggregation tendencies. Overcoming these hurdles will also require additional work on optimizing pharmacokinetics, scaling up production, and integrating the probe with intraoperative navigation systems. Despite these challenges, this work represents a promising advancement in precision oncology, with the potential to improve breast cancer diagnosis and treatment in the future.

## CRediT authorship contribution statement

**Chunting Wang:** Conceptualization, Data curation, Formal analysis, Investigation, Writing – original draft. **Jingjing Hu:** Validation, Visualization. **Yuelin Huang:** Validation, Visualization. **Yanhong Chen:** Validation, Visualization. **Ling Zhan:** Validation, Visualization. **Huanhuan Liu:** Conceptualization, Formal analysis. **Defan Yao:** Project administration, Supervision, Writing – review & editing. **Dengbin Wang:** Funding acquisition, Project administration, Supervision.

## Declaration of competing interest

The authors declare that they have no known competing financial interests or personal relationships that could have appeared to influence the work reported in this paper.

## Data Availability

Data will be made available on request.
