# Supplementary material for: FAP-targeting peptide-directed nanoprobes enable tumor microenvironment-activatable MR/NIRF imaging of breast cancer primary tumor and lung metastases
Source: Mater Today Bio. 2026 Mar 31;38:103064. doi: 10.1016/j.mtbio.2026.103064 (PMC13091518; doi:10.1016/j.mtbio.2026.103064)
Supplement: Multimedia component 2 [file mmc2.docx]

**FAP-Targeting Peptide-Directed Nanoprobes Enable** **Tumor Microenvironment-Activatable MR/NIRF Imaging of Breast Cancer Primary Tumor and Lung Metastases**

Chunting Wang^1, 3#^, Jingjing Hu^1, 2#^, Yuelin Huang^1#^, Yanhong Chen^1^, Ling Zhan^1^, Huanhuan Liu^1*^, Defan Yao^1, 2, 3*^, Dengbin Wang^1, 2, 3^^*^

^1^ Department of Radiology, Xinhua Hospital, Shanghai Jiao Tong University School of Medicine, Shanghai, 200092, China

^2^ Faculty of Medical Imaging Technology, College of Health Science and Technology, Shanghai Jiao Tong University School of Medicine, Shanghai, 200025, China

^3^ Shanghai University of Sport, Shanghai, 200438, China

#These authors contributed equally to this work.

^*^Corresponding authors:

E-mail: Huanhuan Liu, liuhuanhuan@xinhuamed.com.cn; Defan Yao, [yaodefan@xinhuamed.com.cn](mailto:yaodefan@xinhuamed.com.cn); Dengbin Wang, [wangdengbin@xinhuamed.com.cn](mailto:wangdengbin@xinhuamed.com.cn)


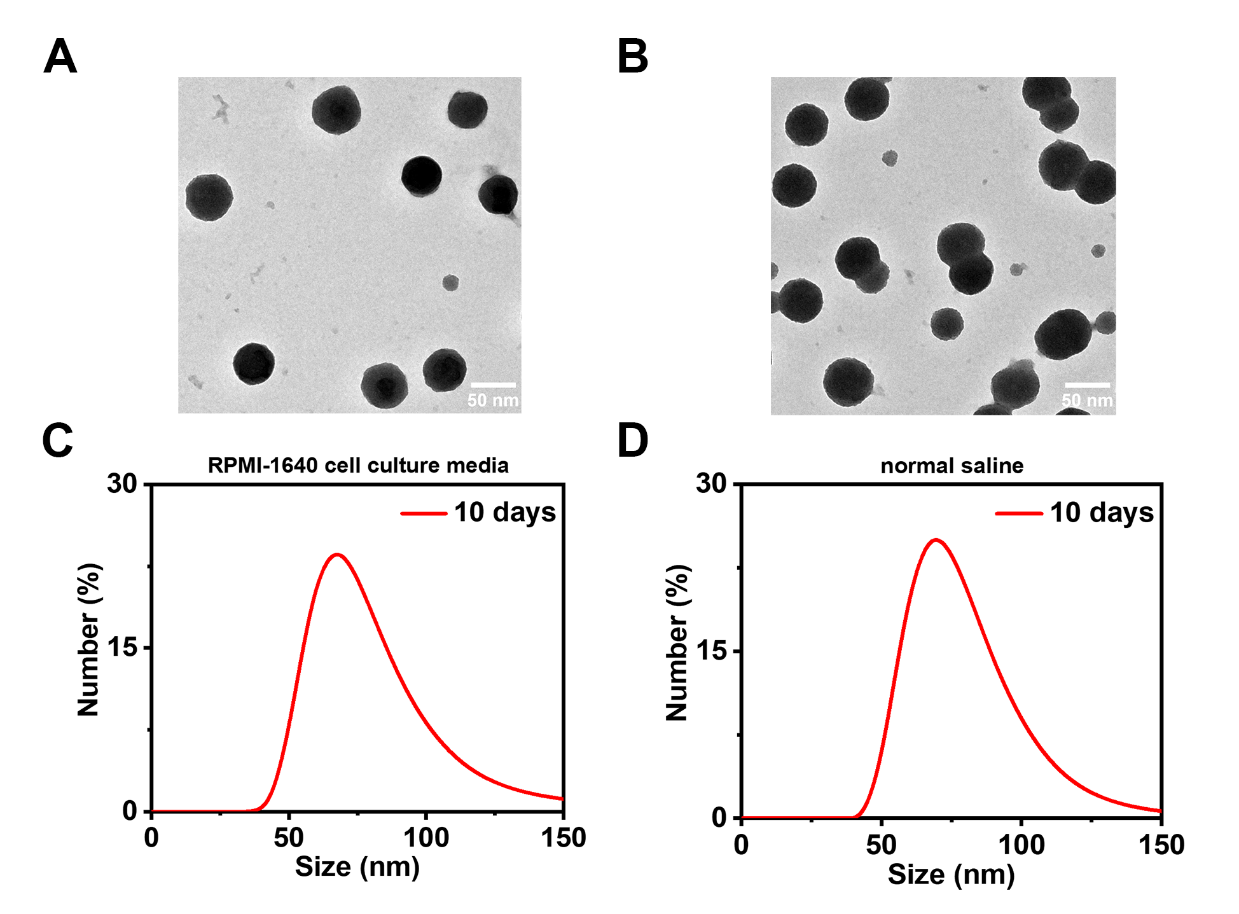


**Figure S1.** Stability of FAP-Cy7@MnO₂ nanoparticles in RPMI-1640 media and normal saline. (A, B) TEM images of FAP-Cy7@MnO₂ nanoparticles incubated for 10 days in RPMI-1640 media and normal saline, respectively. (C, D) DLS analysis of FAP-Cy7@MnO₂ nanoparticles incubated for 10 days in RPMI-1640 media and normal saline, respectively.

**Figure S2.**TEM mapping of FAP-Cy7@MnO₂ nanoparticles. (A) pH=7. (B) pH=6.5.

**
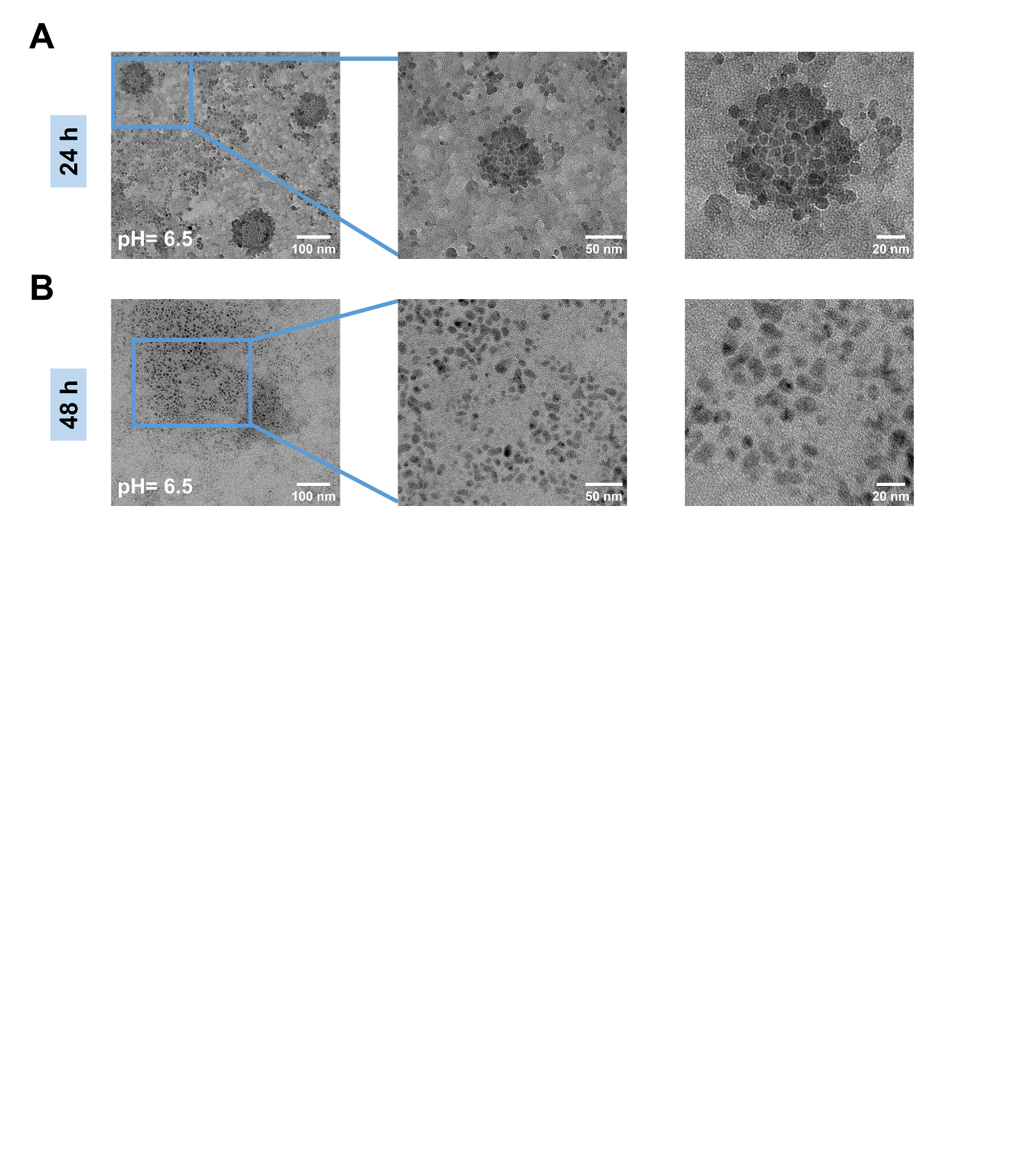
**

**Figure S3.** TEM images of FAP-Cy7@MnO₂ at pH 6.5, showing changes at different time points. (A) 24 h. (B) 48h.

**Figure S4.** DLS analysis of FAP-Cy7@MnO₂ at different pH levels (pH=6.5, 24h/48h), illustrating size variations.

**Figure S5.** The release profiles of manganese ion in PBS buffers (37 °C) at pH 7.4 and 6.5.

**Figure S6.** Absorption spectra of FAP-Cy7@MnO₂ nanoparticles at different concentrations.

**Figure S7.** Fluorescence spectra of FAP-Cy7@MnO₂ nanoparticles at a concentration of 10 μM after incubation for 24 hours under pH 7.4 and 6.5 conditions.


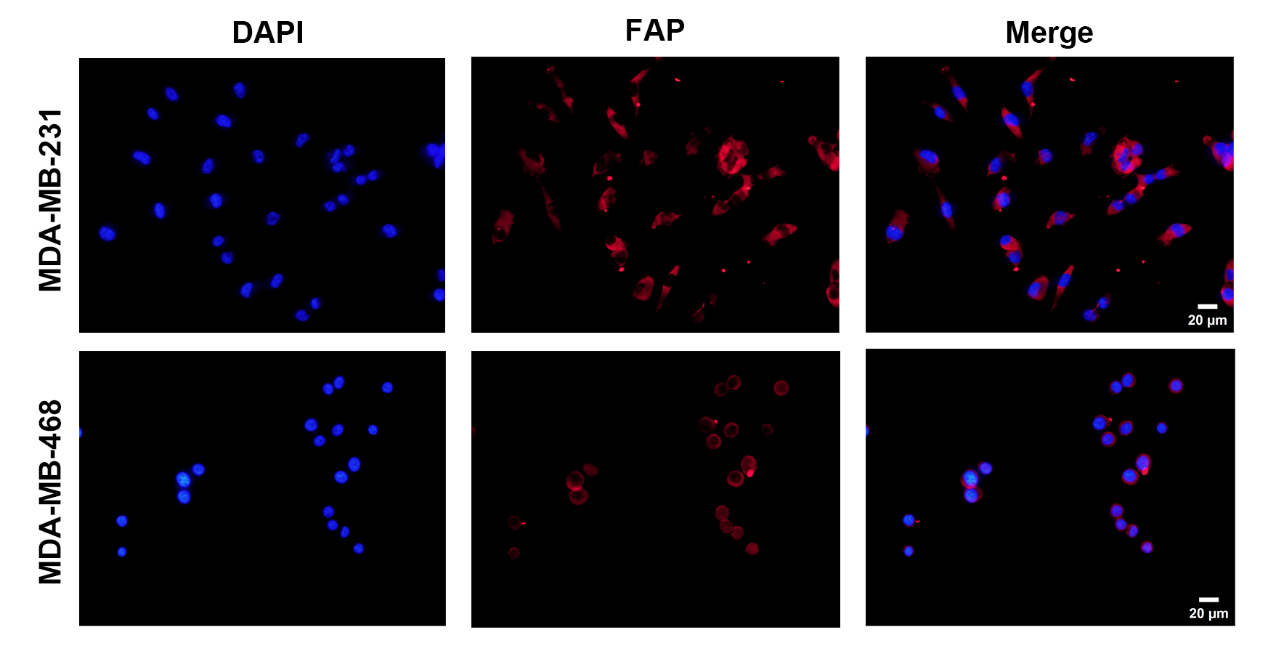


**Figure S8.** Representative immunofluorescence images of FAP expression in MDA-MB-231 and MDA-MB-468 cells. Scale bar: 20 μm.


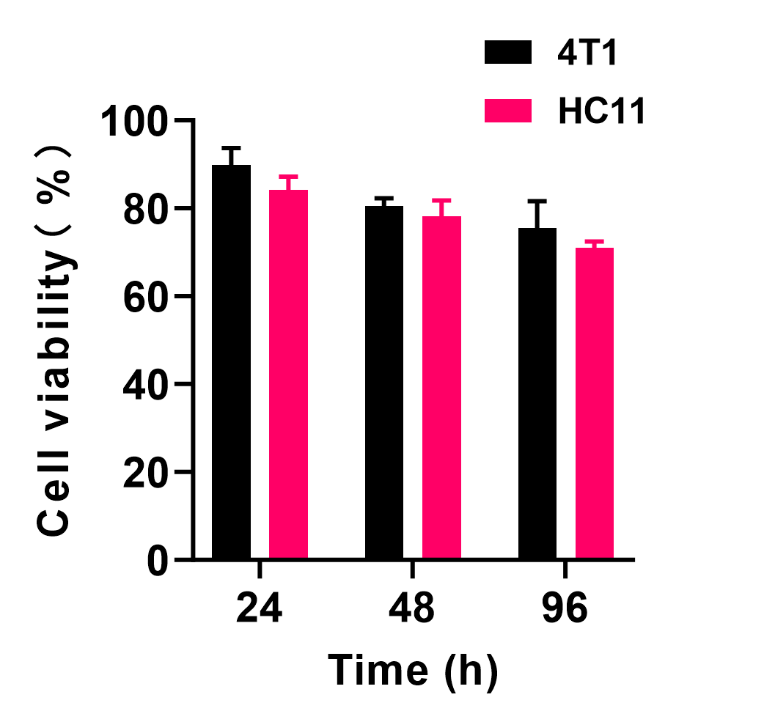


**Figure S9.** Cell viability assay of 4T1 and HC11 cells incubated with FAP-Cy7@MnO₂ nanoprobes at 128 µM for different durations.


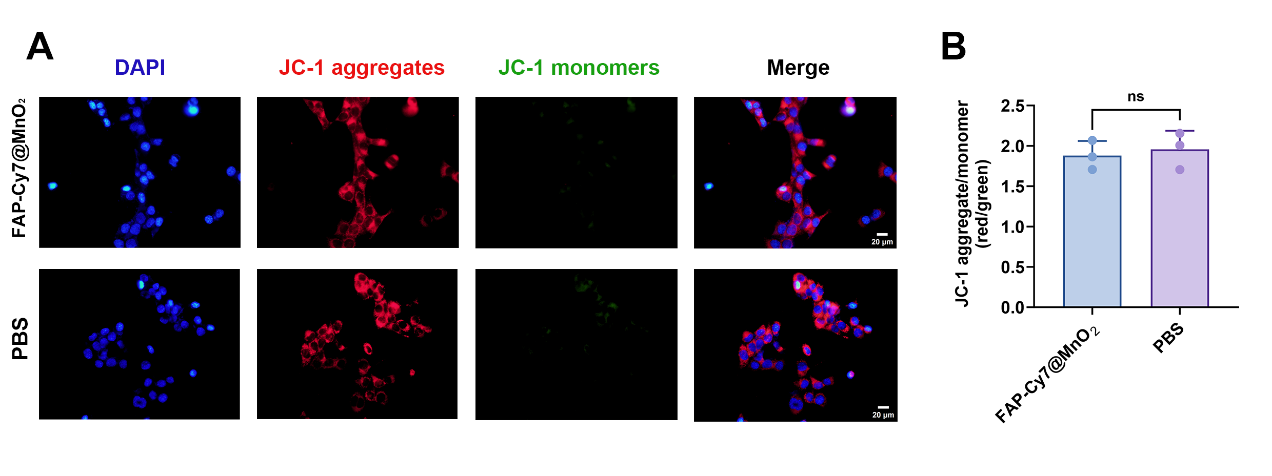


**Figure S10.** (A) JC-1 fluorescence images of 4T1 cells treated with PBS or FAP-Cy7@MnO₂. Scale bar: 20 μm. (B) Quantitative analysis of JC-1 fluorescence intensity under different conditions.

**
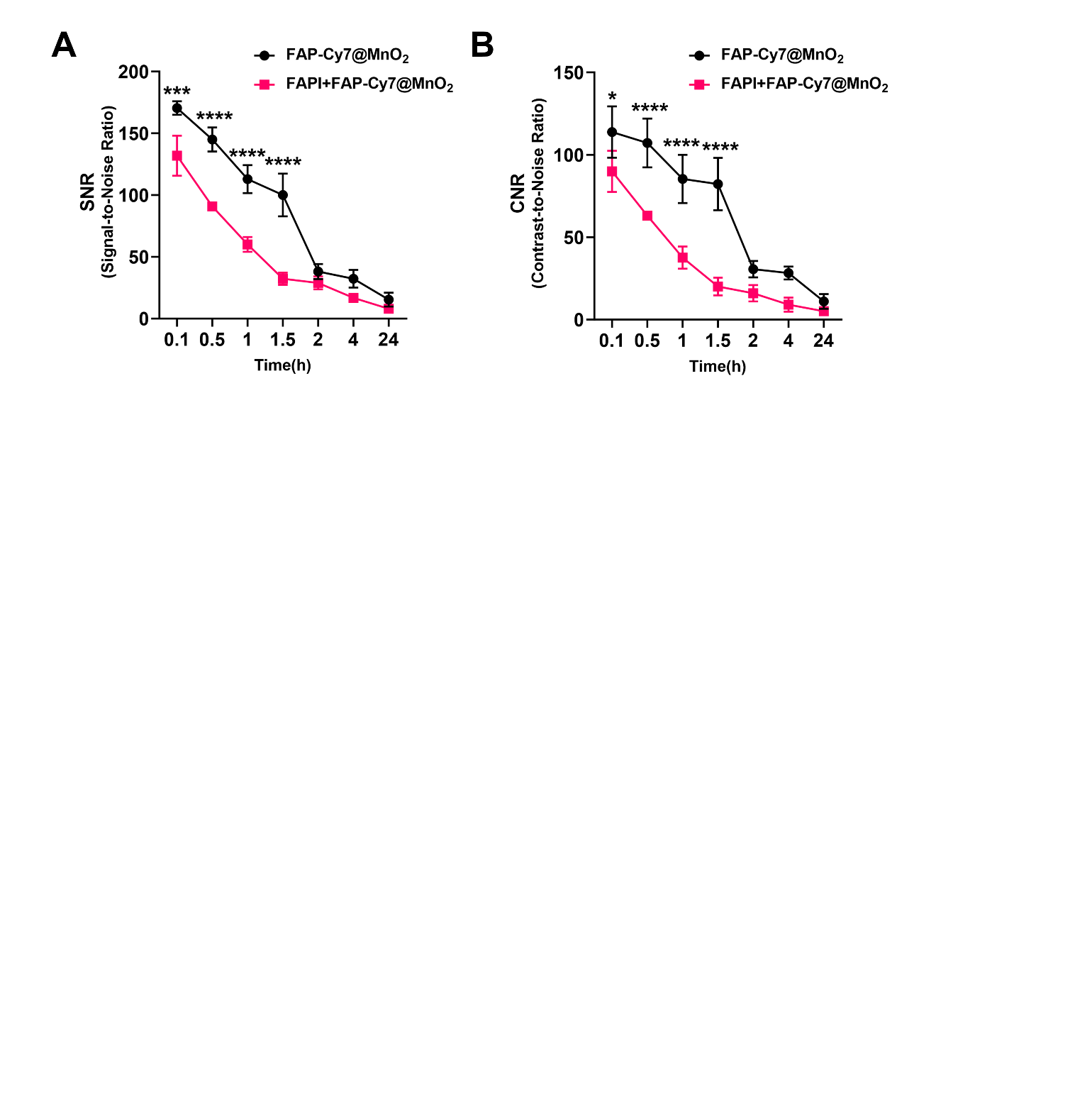
**

**Figure S11.** (A-B) Quantitative time-dependent fluorescence intensity changes in SNR and CNR of NIRF images following the injection of FAP-Cy7@MnO₂, FAPI+FAP-Cy7@MnO₂ (*p < 0.05, ***p < 0.001, ****p < 0.0001).

**
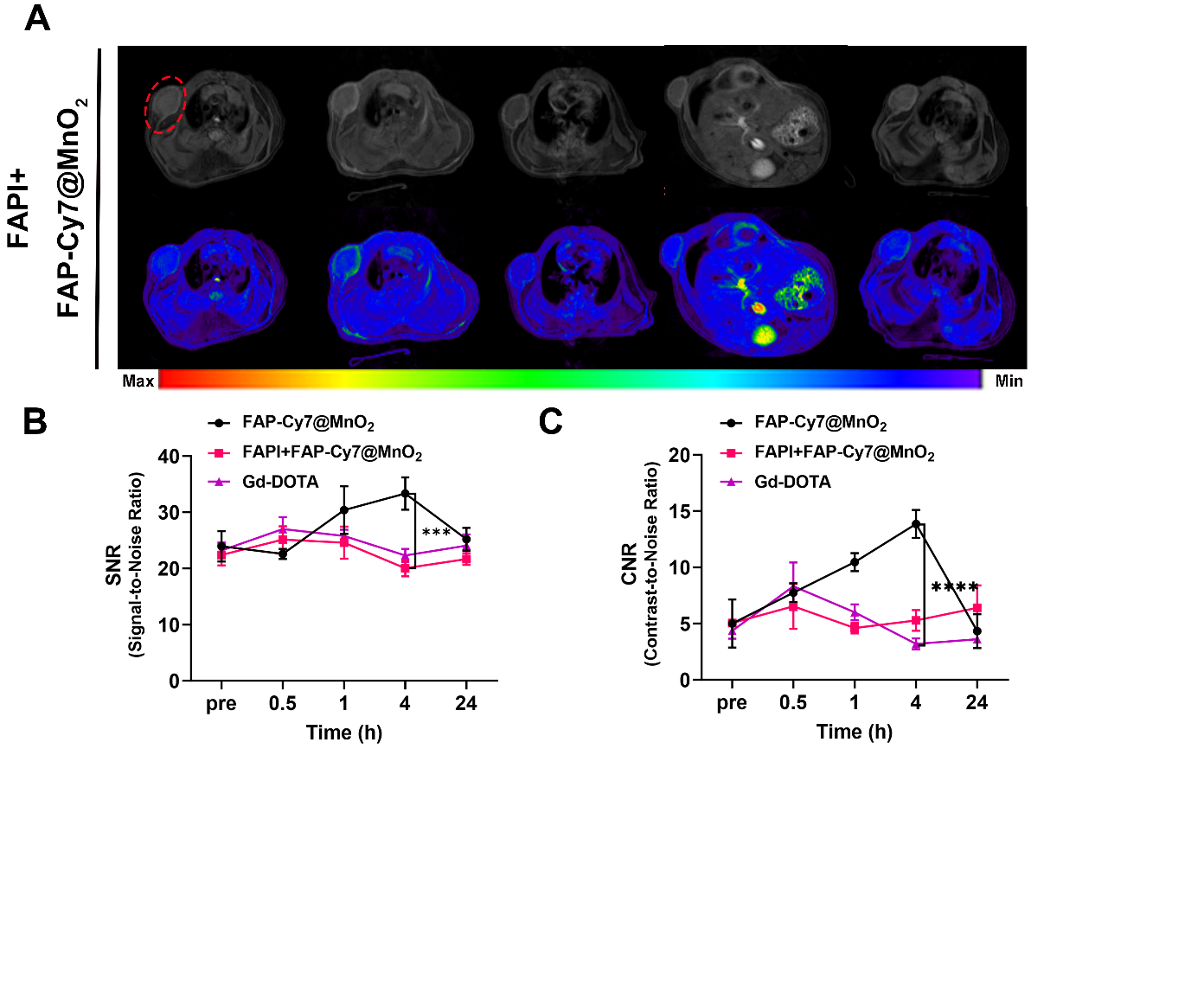
**

**Figure S12.** (A) Representative in vivo T_1_-weighted MR images following the injection of FAPI+FAP-Cy7@MnO₂. (B-C) Quantitative time-dependent changes in SNR and CNR of MRI following the injection of FAP-Cy7@MnO₂, FAPI+FAP-Cy7@MnO₂, or Gd-DOTA (***p < 0.001, ****p < 0.0001).


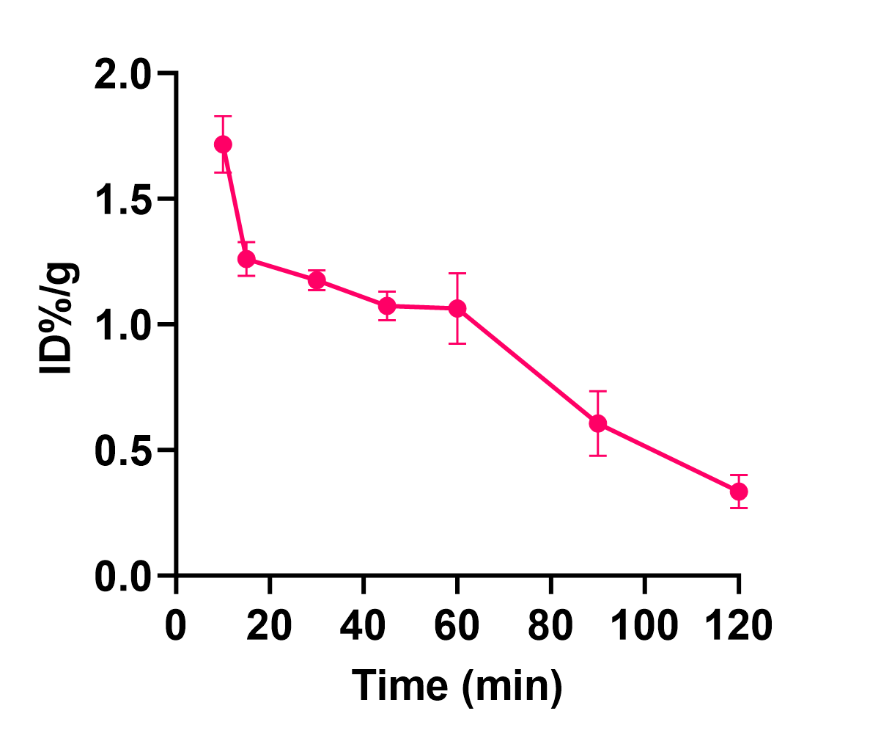


**Figure S13.** ICP-MS analysis of manganese ions in plasma following tail vein injection of FAP-Cy7@MnO₂ in mice. Data are presented as mean ± standard deviation (n=3).

**Figure S14.** Bioluminescence images of lung metastasis nodules in mice.

**Figure S15.** Hemolytic activity of different concentrations of FAP-Cy7@MnO_2_ on erythrocytes.


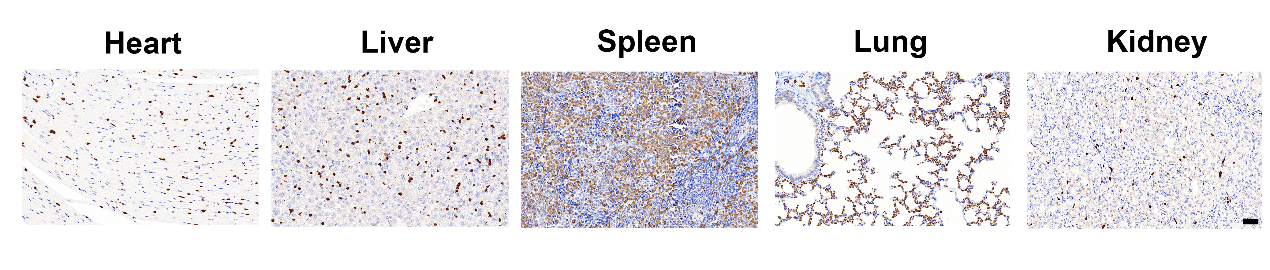


**Figure S16.** Anti-FAP immunohistochemical staining of major organs. Scale bars: 20 µm
